# Exploring the Prevalence and Predictors of Anxiety among Lithuanian Adolescents during Times of Crisis: A Cross-Sectional Study

**DOI:** 10.3390/children11010032

**Published:** 2023-12-27

**Authors:** Laura Šalčiūnaitė-Nikonovė, Monika Žemaitaitytė, Kastytis Šmigelskas

**Affiliations:** 1 Health Research Institute, Lithuanian University of Health Sciences, LT-47181 Kaunas, Lithuania; 2 Department of Health Psychology, Lithuanian University of Health Sciences, LT-47181 Kaunas, Lithuania

**Keywords:** anxiety, adolescent, COVID-19, RUW, war, HBSC

## Abstract

Recent events in Europe, like the COVID-19 pandemic and the 2022 Russo–Ukrainian War (RUW), might have sparked anxiety among adolescents. This study aimed to compare anxiety levels in Lithuanian adolescents post-COVID-19 peak (Study 1, October 2021) and during the onset of the RUW (Study 2, April–June 2022). Data from 459 participants in Study 1 and 6637 in Study 2, aged 11–17, were collected through HBSC pilot and national surveys in Lithuania. Self-reported questionnaires assessed anxiety, social media use, stress, loneliness, self-efficacy, and peer support factors. Analysis was conducted using multivariable logistic regressions. Notably, anxiety prevalence in Lithuanian adolescents showed no significant difference between Study 1 and Study 2, stabilizing around 24%. In 2021, stress (OR = 5.89, 95% CI 3.11–11.17), problematic social media use (OR = 4.58, 95% CI 1.89–10.58), and female gender (OR = 2.87, 95% CI 1.58–5.22) significantly predicted anxiety. By 2022, stress (OR = 3.68, 95% CI 3.14–4.30), loneliness (OR = 2.85, 95% CI 2.43–3.35), and lower self-efficacy (OR = 1.40, 95% CI 1.20–1.60) emerged as important predictors. This study enhances our understanding of adolescent anxiety during crises, emphasizing the urgency of addressing multiple factors to manage and support vulnerable youth.

## 1. Introduction

In recent years, Europe has faced a persistent crisis due to unprecedented events, notably the onset of the global COVID-19 pandemic in early 2020 followed by the 2022 Russo–Ukrainian War. Stressful events like these can increase the levels of psychological distress in the general population [1,2,3], with young people being the significant risk group that requires special attention [2].

Anxiety emerges as a prominent and severe affective symptom among children and adolescents during global crises. While anxiety is a natural response to life’s stressors, events like nearby wars or pandemics can exacerbate it into prolonged or maladaptive states. Over the past decade, studies suggest an increasing trend in the prevalence rates of anxiety disorders in adolescents, varying from 6% [4] to 32% [5]. Anxiety disorders are recognized as one of the most disabling conditions in adolescents [6], causing challenges across different psychosocial domains, including academic and social functioning [7] and overall quality of life [8]. Furthermore, elevated anxiety during adolescence can predict future depression, substance abuse, and adult anxiety disorders [9]. Hence, during crises, it becomes crucial to closely observe anxiety symptoms among young individuals.

The profound impact of the COVID-19 pandemic on adolescent anxiety levels is evident, with reported symptomatology rates ranging from 23% to 38% [10,11], indicating a significant rise in mental health concerns [12]. Various pandemic-related factors, such as enforced social restrictions and extended periods at home, negatively affected adolescents’ overall well-being [13], intensifying feelings of isolation and anxiety [14,15]. School closures disrupted established routines, contributing to an increase in depression cases [13,16]. Parental distress during prolonged homestays emerged as a significant predictor of heightened anxiety in children [11,17]. Moreover, the surge in social media use, up to 10 h daily for some adolescents during the lockdown, was found to be linked to increased anxiety, disrupted sleep patterns [18,19,20], and heightened depression prevalence, especially among girls [19]. Finally, misinformation on social media notably added to heightened anxiety, particularly for those struggling to verify information accurately [13,18,21].

The Russo–Ukrainian War, commencing in February 2022, may have further impacted teenagers’ mental health, particularly anxiety. While Ukrainians, especially children, directly experience the war’s impact, its ongoing nature could heighten anxiety across Europe, notably affecting neighboring regions, as seen in similar events, like the Chornobyl disaster [22,23,24]. Lithuania’s historical experiences with Russian occupation in the 20th century may heighten susceptibility to similar situations, fostering increased feelings of threat and insecurity.

Although the scientific literature examining the direct impact of the Russo–Ukrainian War on adolescent populations remains scarce, there have been studies analyzing university students, providing valuable insights. Reports from Czech Republic universities indicated a significant rise in war-related anxiety levels among students [25,26], with older age and female gender correlating with higher anxiety rates [25,27]. Proximity to conflict zones, as observed in Slovakian students compared to their Czech counterparts, seems to exacerbate these concerns [26]. Social media, akin to its role during the pandemic, notably magnifies anxiety. Platforms like Instagram and TikTok may contribute to heightened anxiety from war-related exposure, echoing past instances where media exposure contributed to increased anxiety [28]. Indeed, recent studies highlighted a correlation between media coverage of the RUW and peritraumatic dissociative experiences and anxiety in adolescents [27,29].

Given the importance of recent events on adolescents’ well-being and the current scarcity of research, this study seeks to assess anxiety levels among Lithuanian adolescents following the peak of the COVID-19 pandemic (October 2021) and the initiation of the conflict in Ukraine (April–June 2022). Additionally, this research aims to establish potential mental health and behavioral factors associated with anxiety outcomes, with a particular focus on variables such as excessive social media engagement, feelings of loneliness, stress, self-efficacy, and other putative factors. Finally, this study seeks to determine whether discernible differences in anxiety levels exist between cohorts or whether distinct traits are prevalent within each group. By exploring these differences, it intends to highlight the specific impact of varied events on anxiety and ascertain the potential existence of shared contributing factors across these unique circumstances.

## 2. Materials and Methods

### 2.1. Study Process and Sample

Data for this study were collected from Lithuanian schools at two different time points: during the HBSC national pilot survey in December 2021 (Study 1) and during the HBSC main national survey in Lithuania conducted between April and June 2022 (Study 2).

**Figure 1:**
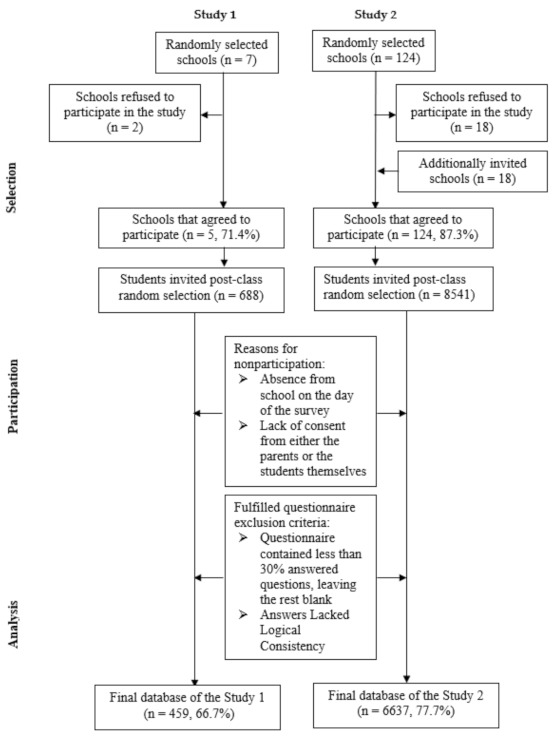
Consort diagram illustrating sample selection for Study 1 and Study 2.

Study 1 was conducted as a pilot project for an upcoming 2022 HBSC study in Lithuania. Seven schools were randomly selected from the national schools’ list (two from the largest city, two from other cities, and three from towns) by choosing the first five schools that agreed to participate in the study (school response rate 71.4%). Of the 688 students invited, 459 participated in the survey (student response rate 66.7%). A consort diagram of the study sample is presented in Figure 1.

During the main national survey (Study 2) performed to compose the geographically representative sample, a total list of general education and vocational schools in Lithuania was stratified by 15 regions (5 large city municipalities and 10 counties, except for large cities), and then 142 schools were randomly selected for participation in this study. Of them, 124 agreed to participate (school response rate 87.3%). The eligible sample in the agreed-to-participate schools was 8541 students, but some students did not attend school on the day of the survey, refused to participate, or returned corrupt questionnaires, resulting in 6637 students being included in the analysis (student response rate 77.7%) (Figure 1).

In accordance with the HBSC study protocol, the questionnaire was administered in every school to students in the 5th, 7th, 9th, and 11th grades, corresponding to the predominant ages of 11, 13, 15, and 17 years, respectively [30]. The 2021 and 2022 samples followed the same protocol requirements, so the distribution of study participants by gender and class was similar. 

### 2.2. Measures

Paper-based self-report questionnaires were used following the internal protocol recommendations of the HBSC study [30]. For the current analysis, the selected indicators were anxiety, problematic social media use, stress, loneliness, self-efficacy, peer support, and sociodemographic indicators such as gender, grade, and family socioeconomic status.

Anxiety was assessed using the seven-item Generalized Anxiety Scale 7 (GAD-7) [31]. Study participants were asked to rate seven anxiety symptoms over the past two weeks on a 4-point scale ranging from 0 ‘not at all’ to 3 ‘almost every day’. Responses were summed and grouped into two categories: (1) no–mild anxiety (0–10 points) and (2) moderate–severe anxiety (11–21 points) [30,32]. The internal consistency of the scale in Study 1 was α = 0.865, and in Study 2 it was α = 0.889.

Problematic social media use was assessed using the nine-item Social Media Disorder (SMD) scale with dichotomous (no/yes) response options [33]. Research participants who answered positively to at least 6 statements were coded as problematic social media users (PSMUs). The internal consistency of the scale in Study 1 was α = 0.813, and in Study 2 it was α = 0.770.

Stress was assessed using the four-item Cohen Perceived Stress Scale (PSS-4) [34]. Respondents rated how often they experienced stressful situations on a 5-point Likert scale ranging from ‘never’ to ‘very often’ in the last month. The PSS-4 is used as a continuous scale (sum score after reverse-coding the 2nd and 3rd items), where higher scores reflect higher levels of perceived stress. In this study, responses were summed and grouped into two categories: (1) low-level stress (0–8 points) and (2) high-level stress (9–16 points). The internal consistency of the scale in Study 1 was α = 0.566, and in Study 2 it was α = 0.590.

Loneliness was measured by asking students how often they felt lonely in the past 12 months using a 5-point scale ranging from ‘never’ to ‘always’ [35]. The answers of the subjects were divided into 2 groups: ‘high level’, those who indicated that they always or mostly feel lonely, and ‘low’, those who indicated that they never, rarely, or sometimes feel lonely.

Self-efficacy was measured using two questions about problem solving and decision implementation. Study participants were asked to rate their abilities on a 5-point scale ranging from ‘never’ to ‘always’. Their responses were divided into 2 groups: ‘high’, those who indicated that they always or most of the time managed things successfully, and ‘low’, those who indicated that they never, rarely, or sometimes managed things successfully [30].

Peer support was assessed using one subscale from the Multidimensional Scale of Perceived Social Support (MSPSS) [36]. Study participants were asked to rate their answers on a 7-point Likert scale ranging from ‘very strongly disagree’ to ‘very strongly agree’. High peer support was defined as a mean score of 5.5, indicating high peer support [30]. The internal consistency of the scale in Study 1 was α = 0.913, and in Study 2 it was α = 0.912.

Family socioeconomic status was assessed using the Family Affluence Scale (FAS III), comprising items that reflect market forces, economic trends, and technological advances, as well as cultural, social, and geographical norms in consumption across Europe [37,38]. The FAS III was used as a continuous scale, consisting of six items about owning your private bedroom, family car, computer, dishwasher, bathrooms, and family vacations abroad. Higher scores reflect higher family affluence (the sum score could reach from 0 to 13 points). The internal consistency of the scale in Study 1 was α = 0.449, and in Study 2 it was α = 0.519.

### 2.3. Ethical Considerations

Both studies were approved by the Bioethics Centre of the Lithuanian University of Health Sciences (Study 1 No. BEC-M-03, 24 November 2021; Study 2 No. BEC-M-05, 17 March 2022). Permission was sought from the authorities of each selected school to conduct the research within their premises. Two forms of informed consent were implemented in the study. First, passive written informed consent was utilized, where parents were informed about their children’s participation through the school administration. The school administration was instructed to inform parents about the upcoming survey through electronic diaries or other communication methods preferred by the school. Parents had to notify the administration of their refusal to participate. Second, active verbal consent was obtained from the adolescents before the survey commenced. At the beginning of the study, the researchers presented the purpose of the study, the principles of confidentiality and anonymity, and the option to refuse participation. The anonymity of the research participants and the confidentiality of the data were ensured. The data were collected by trained researchers.

### 2.4. Data Analysis

Data were processed using MS Excel 2016 and analyzed using IBM SPSS Statistics for Windows, Version 28.0 [39]. A statistical significance level of α = 0.05 (*p* < 0.05) was used. Cronbach’s alpha coefficient was calculated to assess the internal consistency of the scales.

Pearson’s chi-squared test was used to compare the prevalence of anxiety by gender, class, and other dichotomized psychological factors between the 2021 and 2022 cohorts. Student’s *t*-test was used to compare family socioeconomic status (FAS III) means between anxiety groups. The main analysis to identify prognostic factors of anxiety was conducted using univariate and multivariable binary logistic regressions. The strength of associations was expressed in odds ratios (ORs) with 95% confidence intervals (CIs), while the model fits were assessed using Nagelkerke R^2^.

## 3. Results

Detailed characteristics of the study sample are presented in Table 1. In both cohorts, the gender distribution aimed for parity at around 50 percent. Among the grades, 9th grade participation was slightly higher, constituting approximately 28–29 percent, while 11th grade involvement was the lowest, at around 21 percent. The average Family Affluence Scale (FAS) score ranged between 7 and 8 points.

The descriptive results in Table 2 show that the prevalence of anxiety among adolescents in 2021 and 2022 had no statistically significant difference (*p* = 0.515). Almost a quarter of adolescents (23–24%) in the 2021 and 2022 groups had elevated levels of anxiety on the GAD-7 scale, indicating a clinically significant risk of anxiety.

After comparing anxiety levels based on demographic and social characteristics, a significant gender disparity appeared, with girls expressing moderate or severe anxiety at about double the prevalence of boys (33–34% compared to 14–15%). Statistically significant gender differences were evident in both the 2021 and 2022 cohorts (*p* < 0.001). Furthermore, the results revealed systematic differences related to age, indicating a higher prevalence of elevated anxiety among older adolescents. Notably, a statistically significant increase in anxiety levels was observed in the 2022 cohort when comparing 5th and 11th grade students. Additionally, a higher risk of anxiety was related to lower socioeconomic status in 2021 and 2022, although no statistically significant differences were observed (*p* = 0.066 and *p* = 0.293, respectively).

Prior to developing prognostic anxiety models, it was important to examine potential variations in psychological factors among the examined cohorts (Table 3). This step aimed to comprehend any inherent differences or similarities between the groups, recognizing their potential influence on the manifestation and experience of anxiety within each cohort. Subsequently, the observed cohorts did not display statistically significant differences when evaluating other psychological factors (*p* > 0.05). This study showed that almost one-third of Lithuanian teenagers (28–31%) experienced heightened stress, and one-fifth (20–22%) reported feelings of loneliness. Furthermore, one in ten adolescents (10–12%) exhibited a risk for problematic social media use. Additionally, more than 40% of young people expressed a perceived lack of peer support, and approximately half of the surveyed adolescents (49–53%) assessed their self-efficacy as low in problem solving and decision implementation.

Finally, to investigate the potential relationship between anxiety and selected psychological factors within the 2021 cohort, univariate regression analyses were conducted (Table 4). Substantial associations emerged between anxiety and specific factors. Notably, high stress levels (OR = 6.46, 95% CI 3.96–10.51), problematic social media use (OR = 3.75, 95% CI 1.97–7.13), and female gender (OR = 3.12, 95% CI 1.9–5.03). High loneliness levels (OR = 3.82, 95% CI 2.31–6.33), low peer support (OR = 1.71, 95% CI 1.08–2.69), and low self-efficacy in decision implementation (OR = 1.97, 95% CI 1.24–3.15) demonstrated significant associations with anxiety. However, in the multivariable model, these associations lost statistical significance. Self-efficacy (problem solving), grade, and socioeconomic status (FAS III) were not significantly associated with anxiety in the univariate model.

To investigate whether anxiety-related factors remain significant after controlling for competing indicators, two multivariable binary logistic regression models were created (Table 4) to identify independent variables that predict the likelihood of moderate–severe anxiety in 2021. The created model fit the data well: the likelihood ratio test χ^2^ = 91.21, *p* < 0.001; the Hosmer–Lemeshow test χ^2^ = 9.76, *p* = 0.282; and the Nagelkerke coefficient of pseudo determination was 0.326. The model correctly classified 92.8% without anxiety and 41.8% with moderate–severe anxiety. The overall percentage of correct predictions of the model was 80.2%.

The multivariable regression model predicting anxiety in the year 2021 revealed that high-level stress increased the probability of anxiety in adolescents by 5.89 times (95% CI 3.11–11.17). PSMU demonstrated a substantial effect on anxiety, with an OR of 4.58 (95% CI 1.89–10.58). In this model, girls were at higher risk of experiencing anxiety (OR = 2.87, 95% CI 1.58–5.22). All other indicators did not demonstrate significant associations with anxiety in the multivariable model. Overall, stress, problematic social media use, and gender emerged as significant predictors of anxiety in this cohort, with the strongest factor being stress.

Analyzing the data in the 2022 cohort, all included variables showed a significant association with anxiety in the univariate model (Table 5). The multivariable binary logistic regression model for predicting the likelihood of moderate–severe anxiety in 2022 fit the data well: the likelihood ratio test χ^2^ = 1245.99, *p* < 0.001; the Hosmer–Lemeshow test χ^2^ = 11.29, *p* = 0.186; and Nagelkerke coefficient of pseudo determination was 0.305. The model correctly classified 92.2% without anxiety and 41.0% with moderate–severe anxiety. The overall percentage of correct predictions of the model is 79.5%.

In the 2022 multivariable regression model, heightened stress increased the probability of experiencing anxiety by 3.68 times (95% CI 3.14–4.30), while problematic social media use increased the probability by 1.88 times (95% CI 1.51–2.33). Unlike in 2021, this model confirmed that loneliness significantly increased the probability of anxiety by 2.85 times (95% CI 2.43–3.35), and low self-efficacy (problem solving) also increased the probability of anxiety but was weaker (OR = 1.40; 95% CI 1.20–1.60). Additionally, older teenagers exhibited an increased risk of experiencing anxiety across different grades: 7th (OR = 1.26, 95% CI 1.02–1.57), 9th (OR = 1.33, 95% CI 1.08–1.63), and 11th (OR = 1.43, 95% CI 1.15–1.79). This trend was consistent with the higher likelihood observed among girls (OR = 2.26, 95% CI 1.94–2.62) and adolescents from lower socioeconomic status families (OR = 1.07, 95% CI 1.04–1.11).

Thus, multivariable models suggest that high levels of stress, problematic social media use, and female gender were consistent independent predictors of anxiety in the 2021 and 2022 cohorts. For these indicators, the effect sizes in terms of odds ratios were larger in 2021 compared to 2022. In contrast, loneliness and self-efficacy (problem solving) in 2022 were stronger predictors for anxiety compared to 2021.

## 4. Discussion

Anxiety is a common emotional state among children and adolescents during global crisis periods [4]. The COVID-19 pandemic and the Russian invasion of Ukraine have heightened global tensions, potentially leading to increased anxiety levels. Nevertheless, research on adolescent mental health during these difficult years, particularly in northeastern Europe, remains limited. Therefore, this study aimed to assess anxiety levels in Lithuanian adolescents during two key timeframes: October 2021 (marking the second COVID-19 wave) and April–June 2022 (coinciding with the onset of the Russian invasion of Ukraine). Furthermore, we have examined the potential influence of various psychological factors, including loneliness, problematic social media use, stress, and self-efficacy, on anxiety levels during these specific time periods.

Our research revealed that around 23–24% of adolescents in 2021 and 2022 showed elevated anxiety levels, indicating a clinically significant risk of anxiety. Notably, within the 2022 cohort, this increase was particularly notable among older students. Although there was no statistically significant difference in anxiety rates between 2021 and 2022, overall trends suggest higher rates compared to previous studies during calmer periods, where adolescent anxiety levels were typically around 6% [4]. Similar findings were noted in recent Finnish research examining trends in generalized anxiety among adolescents from 2013 to 2021 using the GAD-7 questionnaire. From 2013 to 2019, anxiety levels for males aged 13 to 17 years ranged between 5 and 6%, and for females in the same age group, it varied from 15 to 20% [40]. However, in 2021, anxiety levels increased to 7% for males and 30% for females. In our study, female participants showed a tendency to experience heightened anxiety more than twice as often as their male counterparts, both in 2021 and 2022. This difference remained statistically significant, even after adjustment for additional factors in the models (OR 2.87 and 2.26, respectively).

We found that around one-third of Lithuanian adolescents felt heightened stress levels, which may also lead to increased anxiety. Indeed, in our study, stress has demonstrated significant correlations with anxiety in univariate regressions and appeared as the most influential component in both the 2021 and 2022 multivariable regression models, with odds ratios of 5.9 and 3.7, respectively. This suggests that students experiencing higher stress levels were 4–6 times more likely to experience anxiety, independent from other included factors. This negative impact of high perceived stress levels on increasing anxiety, depression, and other mental health risk factors has been documented in prior research [41,42]. Feelings of a lack of control, a belief that one cannot effectively manage emerging challenges, and an excessive internal locus of control can trigger anxiety and fear of unpredictable events. In recent years, this connection between anxiety and stress among young people may have been more pronounced than before. The COVID-19 pandemic, as supported by other studies, increased stress and subsequently anxiety levels by disrupting the daily routines of young individuals, limiting social interactions and intensifying feelings of isolation, thereby raising concerns about virus transmission [43,44]. Furthermore, with the onset of the conflict in Ukraine, the stress levels of neighboring countries escalated even further. Concerns about the recent war in Ukraine were found to have increased in Polish [45], Romanian [27], and Czech youth [26]. The frequency of news consumption during times of military conflict played a significant role in exacerbating stress and anxiety [26,46,47]. Broadcasting the unpredictability of the conflict’s outcome, potential threats to other countries, and witnessing death and violence could further lead to secondary trauma.

Hence, social media, recognized as a primary source for accessing news in the contemporary world, particularly among the youth, has been also identified as a potential contributor to heightened anxiety levels in this research. In our study cohorts, problematic social media use was evident in 11.5% (in 2021) and 10.3% (in 2022) of young individuals, with no significant difference between cohorts. PSMU has also emerged as a significant predictive factor for anxiety in our samples. According to logistic regression models, youths exhibiting problematic social media use were associated with 4.9 times higher odds of experiencing elevated anxiety in 2021 and 1.9 times higher odds in 2022. Prior research has shown that PSMU is linked to sleep disturbances and reduced life satisfaction [48], as well as depression, anxiety, and stress [49]. Various hypotheses can be formulated regarding the connection between PSMU and anxiety during these periods, including higher anxiety due to exposure to distressing news on social media [25], the ‘fear of missing out’ [50], or the possibility that the addictive behavior itself is associated with anxiety (the inability to stay away from social media, which is one of the symptoms of PSMU, may trigger anxiety). On the other hand, it is important to note that our study was cross-sectional, and the relationship between anxiety and PSMU may be bidirectional—individuals experiencing anxiety may be more prone to engage in PSMU [51].

Almost half of the surveyed adolescents reported low self-efficacy which, in turn, increased odds of experiencing anxiety. In our study, self-efficacy was examined by two items related to problem solving and decision implementation, but only the former exhibited statistical significance within the multivariable regression model in 2022. We found that adolescents who think that they usually cannot find a solution to an emerging challenge exhibit a 1.4-fold increased likelihood of experiencing heightened anxiety. Similar results demonstrating links between low self-efficacy and anxiety were observed in previous studies [52,53]. Interestingly, the question about decision implementation in our study demonstrated statistical significance in bivariate analysis but lost this significance when controlling for other variables. This suggests that the link between increased anxiety and self-efficacy is more related to how individuals perceive their ability to come up with solutions rather than whether they put those solutions into practice. Hence, the affective dimension of decision-making assumes greater salience. It is worth noting that such an association did not manifest within the 2021 cohort.

Our research findings indicated that 20–22% of the surveyed students reported feeling lonely most of the time. Over the past decade, there has been a rapid increase in loneliness among young people [54]. This trend could have been accelerated further by the onset of the global COVID-19 pandemic, which imposed social restrictions and may have increased the number of people feeling lonely [55]. In our study, loneliness was also significantly linked to increased levels of anxiety. In 2022, students who reported feeling lonely were nearly three times more likely to experience heightened anxiety, even after controlling for other factors. A similar trend was noted in the 2021 cohort, although the associations did not reach statistical significance. These results were in line with the recent literature review revealing that higher levels of loneliness during the COVID-19 pandemic were significantly associated with poorer well-being, including higher depression symptoms, anxiety symptoms, gaming addiction, and sleep problems [56].

Peer support was another factor that was analyzed in our study. We found that low peer support was present in slightly more than 42% of both study cohorts, and there was no statistically significant difference between years. Low social support showed significant correlations with higher anxiety levels in univariate regressions; however, when other factors were included in the models, these associations became non-significant. Research indicates that peer support is one of the most significant factors, after parental support, which can influence a child’s psychological well-being [57]. Peer conflicts, victimization, or isolation can increase suicidal ideation and anxiety [58]. One possible explanation for the lack of the significance of peer support in multivariable regression models is the inclusion of loneliness as a variable, which may reduce the role of social support in predicting anxiety. Indeed, studies suggest that loneliness serves as a mediator between social support and life satisfaction: when a person has social support, they feel less lonely, and as a result, their life satisfaction increases [59].

Upon summarizing both studies, it became evident that anxiety levels remained comparable between 2021 and 2022, aligning with the respective prognostic factors. This similarity may be attributed to the assessment of generalized anxiety disorder symptoms using the GAD-7 questionnaire rather than focusing solely on event-specific anxiety. Such an observation suggests that a general anxiety assessment could yield a similar predictive model across both periods. However, the multivariate regressions revealed variations in the impact degrees of prognostic factors. In 2021, high stress, social media use, and female gender emerged as more prominent predictors. Conversely, in 2022, loneliness and problem solving self-efficacy became stronger predictors for anxiety compared to 2021. Several hypotheses could be derived from these findings. Plausibly, COVID-19 might have exerted a more substantial influence on anxiety levels than the impact of war during the respective periods. Additionally, the year 2022 possibly witnessed a heightened adaptation to the persistent threat of COVID-19. Factors contributing to this adaptation include the prolonged duration since the pandemic’s onset, advancements in disease control through vaccination and treatment, the facilitation of open school settings, and a shift in public focus toward the war. These adjustments potentially influenced the behavioral dynamics of predictive factors associated with anxiety, thereby contributing to the observed differences in odds ratios. Nevertheless, a comprehensive exploration of these hypotheses necessitates longitudinal study designs. Such designs would enable a more detailed investigation into the causal relationships and temporal dynamics between predictive factors and anxiety levels across varying timeframes.

There are several limitations of our research that need to be addressed. First, the cross-sectional nature of this study restricts the ability to establish causal relationships between analyzed variables. Therefore, it is important to emphasize that regression models, while predicting anxiety, may also exhibit some reverse or bidirectional associations between dependent and independent variables. The main aim of our study was to explore the anxiety rates and their correlates among Lithuanian adolescents during two distinct time periods: the second wave of the COVID-19 pandemic and the onset of the Russian invasion of Ukraine. However, it is essential to acknowledge that both studies were separated by half a year, which implies that residual COVID-related influences on overall anxiety levels and other indicators may have persisted within the 2022 cohort as well. On the other hand, it is worth noting that the potential lasting effects of the COVID-19 pandemic may remain relevant in the years to come, making the elimination of this background influence a challenging task. It is also noteworthy that the examined cohorts had considerably different sample sizes. While statistically significant associations between the psychological characteristics of cohorts were not found, the statistical significance in the regression models of the 2021 cohort could likely have been the result of a smaller sample size. Finally, our study exclusively incorporated self-report questionnaires. More objective assessments or data from diverse sources, such as parents, teachers, and healthcare professionals, were not collected. Therefore, the findings regarding anxiety expression should be approached with caution. On the other hand, previous research has demonstrated the validity and sensitivity of the GAD-7 questionnaire in assessing anxiety risk [32]. Additionally, recent work by Stromájer and colleagues [60] has highlighted discrepancies between objective stress measurements, such as cortisol levels, and subjective evaluations. This discrepancy emphasizes that while objective measures might not always align with individuals’ subjective experiences, subjective tests used to measure anxiety might offer a more accurate indication of anxiety levels and reflect the inner world of adolescents.

## 5. Conclusions

Anxiety represents a common response among adolescents to life’s unpredictability, which may be particularly exacerbated during periods of global upheaval. Our study demonstrated that approximately 23–24% of Lithuanian adolescents experienced significantly elevated anxiety levels during the second wave of the COVID-19 pandemic and the Ukraine conflict. Moreover, we found that the risk of elevated anxiety levels increased overall stress, decreased self-efficacy, loneliness, or problematic social media use. Additionally, we identified that certain demographic characteristics, including female gender, older age, and lower socioeconomic status, contributed to heightened anxiety risk.

This study not only contributes to the theoretical understanding of anxiety but also offers practical insights. Conducted within a school environment, our research unveiled a significant correlation between anxiety-provoking events and their profound impact on the mental well-being of young individuals. This impact often manifests as heightened levels of anxiety, increased stress, and a noticeable dependence on social media. Therefore, this provides a foundation for educational and health institutions to develop prevention and intervention programs supporting adolescent psychological health during periods of global crises or challenging circumstances. In our research, specific vulnerable groups to anxiety emerged, notably girls, older students, and those from lower socioeconomic backgrounds. Targeted attention toward these cohorts is essential within school settings to identify mental health concerns and provide tailored support. Additionally, our study illuminates the complex relationship between anxiety and various other psychological indicators, emphasizing the importance of comprehensive assessments and integrated approaches in designing and implementing psychological interventions.

## Figures and Tables

**Table 1:** Main characteristics of the study sample.

Characteristics		2021 (Study 1) % (*n*) or m (SD)	2022 (Study 2) % (*n*) or m (SD)
Gender			
	Boys	51.6 (237)	51.1 (3353)
	Girls	48.4 (222)	48.9 (3213)
Grade			
	5th	26.3 (120)	25.4 (1687)
	7th	23.9 (109)	23.9 (1584)
	9th	28.1 (128)	29.3 (1947)
	11th	21.7 (99)	21.4 (1419)
FAS III (0–13)		7.06 (2.24)	7.51 (2.32)

FAS III—Family Affluence Scale, showing the family’s socioeconomic status. Valid percentages were calculated without missing responses.

**Table 2:** Anxiety prevalence in the 2021 and 2022 cohorts and demographic comparisons.

		2021			2022			
		Moderate–Severe Anxiety	No–Mild Anxiety		Moderate–Severe Anxiety	No–Mild Anxiety		
		% (*n*)	% (*n*)	*p* ^	% (*n*)	% (*n*)	*p* ^	*p* *
General population		23.0 (100)	77.0 (335)		24.4 (1528)	75.6 (4741)		0.515
Gender								
	Boys	13.6 (30)	86.4 (191)	<0.001	15.1 (480)	84.9 (2696)	<0.001	
	Girls	32.9 (70)	67.1 (143)		33.7 (1022)	66.3 (2009)		
Grade								
	5th	18.9 (21)	81.1 (90)	0.163	20.0 (313)	80.0 (1254)	<0.001	
	7th	19.6 (19)	80.4 (78)		26.1 (390)	73.9 (1103)		
	9th	29.4 (37)	70.6 (89)		25.7 (476)	74.3 (1375)		
	11th	23.7 (23)	76.3 (74)		25.7 (349)	74.3 (1009)		
FAS III		6.67 ± 2.24	7.15 ± 2.22	0.066	7.47 ± 2.38	7.54 ± 2.29	0.293	

^—associations of anxiety rates and demographic characteristics within the 2021 and 2022 cohorts; *—comparison of anxiety rates between the 2021 and 2022 cohorts. FAS—Family Affluence Scale. Valid percentages were calculated without missing responses.

**Table 3:** Comparison of psychological factors between the 2021 and 2022 cohorts.

		2021	2022	*p*
		% (*n*)	% (*n*)	
PSMU				
	Problematic use	11.5 (48)	10.3 (624)	0.423
	Non-problematic use	88.5 (370)	89.7 (5463)	
Stress				
	High level	30.7 (138)	28.4 (1821)	0.292
	Low level	69.3 (311)	71.6 (4588)	
Loneliness				
	High	20.0 (92)	21.7 (1422)	0.394
	Low	80.0 (367)	78.3 (5119)	
Peer support				
	Low	44.5 (196)	42.9 (2746)	0.495
	High	55.5 (244)	57.1 (3658)	
Self-efficacy: problem solving				
	Low	50.8 (232)	48.7 (3186)	0.398
	High	49.2 (225)	51.3 (3353)	
Self-efficacy: decision implementation				
	Low	52.5 (238)	52.0 (3394)	0.824
	High	47.5 (215)	48.0 (3133)	

PSMU—problematic social media use. Valid percentages were calculated without missing responses.

**Table 4:** Regression models predicting risk for moderate–severe anxiety in 2021.

		2021					
		Univariate Regression Models			Multivariable Regression Models		
					Nagelkerke R^2^ *		0.326
		OR	95% CI	*p*	OR	95% CI	*p*
Stress							
	Low level	1.00			1.00		
	High level	6.46	3.96–10.51	<0.001	5.89	3.11–11.17	<0.001
Loneliness							
	Low	1.00			1.00		
	High	3.82	2.31–6.33	<0.001	1.71	0.88–3.32	0.112
Social media use							
	Non-problematic	1.00			1.00		
	Problematic	3.75	1.97–7.13	<0.001	4.58	1.89–10.58	<0.001
Self-efficacy: problem solving							
	High	1.00			1.00		
	Low	1.56	0.99–2.45	0.055	0.62	0.33–1.18	0.146
Self-efficacy: decision implementation							
	High	1.00			1.00		
	Low	1.97	1.24–3.15	0.004	0.95	0.50–1.80	0.949
Peer support							
	High	1.00			1.00		
	Low	1.71	1.08–2.69	0.021	1.55	0.86–2.80	0.148
Gender							
	Boys	1.00			1.00		
	Girls	3.12	1.9–5.03	<0.001	2.87	1.58–5.22	0.001
Grade							
	5th	1.00					
	7th	1.04	0.52–2.08	0.903	1.18	0.49–2.88	0.710
	9th	1.78	0.97–3.28	0.064	2.16	0.98–4.77	0.057
	11th	1.33	0.68–2.60	0.399	1.38	0.58–3.30	0.466
FAS III		0.91	0.82–1.00	0.065	0.99	0.87–1.14	0.949

*—Nagelkerke R^2^ of multivariable regression models. FAS III—Family Affluence Scale.

**Table 5:** Regression models predicting risk for moderate–severe anxiety in 2022.

		2022					
		Univariate Regression Models			Multivariable Regression Models		
					Nagelkerke R^2^ *		0.326
		OR	95% CI	*p*	OR	95% CI	*p*
Stress							
	Low level	1.00			1.00		
	High level	7.06	6.21–8.03	<0.001	3.68	3.14–4.30	<0.001
Loneliness							
	Low	1.00			1.00		
	High	6.05	5.31–6.90	<0.001	2.85	2.43–3.35	<0.001
Social media use							
	Non-problematic	1.00			1.00		
	Problematic	2.96	2.48–3.53	<0.001	1.88	1.51–2.33	<0.001
Self-efficacy: problem solving							
	High	1.00			1.00		
	Low	2.24	1.98–2.52	<0.001	1.40	1.20–1.64	<0.001
Self-efficacy: decision implementation							
	High	1.00			1.00		
	Low	2.24	1.98–2.53	<0.001	1.13	0.96–1.32	0.137
Peer support							
	High	1.00			1.00		
	Low	1.37	1.22–1.54	<0.001	1.09	0.94–1.26	0.246
Gender							
	Boys	1.00			1.00		
	Girls	2.86	2.52–3.23	<0.001	2.26	1.94–2.62	<0.001
Grade							
	5th	1.00			1.00		
	7th	1.42	1.20–1.68	<0.001	1.26	1.02–1.57	0.033
	9th	1.39	1.18–1.63	<0.001	1.33	1.08–1.63	0.007
	11th	1.39	1.16–1.65	<0.001	1.43	1.15–1.79	0.002
FAS III		0.99	0.96–1.01	<0.001	1.07	1.04–1.11	<0.001

*—Nagelkerke R^2^ of multivariable regression models. FAS III—Family Affluence Scale.

## Data Availability

The data presented in this study are available upon request from the corresponding author. The data are not publicly available due to their containing information that could compromise the privacy of research participants.
